# Safety and pharmacokinetic properties of a new formulation of parenteral artesunate in healthy Thai volunteers

**DOI:** 10.1186/s12936-024-05085-9

**Published:** 2024-10-03

**Authors:** Joel Tarning, Borimas Hanboonkunupakarn, Richard M. Hoglund, Kesinee Chotivanich, Mavuto Mukaka, Sasithon Pukrittayakamee, Nicholas P. J. Day, Nicholas J. White, Arjen M. Dondorp, Podjanee Jittamala

**Affiliations:** 1 Mahidol Oxford Tropical Medicine Research Unit, Faculty of Tropical Medicine, Mahidol University, 420/6 Rajvithi Road, Bangkok, 10400 Thailand; 2 Centre for Tropical Medicine and Global Health, Nuffield Department of Medicine, University of Oxford, Oxford, UK; 3 Department of Clinical Tropical Medicine, Faculty of Tropical Medicine, Mahidol University, Bangkok, Thailand; 4 The Royal Society of Thailand, Dusit, Bangkok, Thailand; 5 Department of Tropical Hygiene, Faculty of Tropical Medicine, Mahidol University, Bangkok, Thailand

**Keywords:** Malaria, Pharmacokinetics, Bioequivalence, Formulation, Artesunate, Intravenous, Intramuscular, Healthy volunteer

## Abstract

**Background:**

Parenteral artesunate is the first-line therapy for severe malaria. Artesunate, in its current formulation, must be prepared immediately before administration by first dissolving in sodium bicarbonate solution and then diluting in saline. A novel solvent for rapid and stable single step reconstitution of artesunate was recently developed showing improved solubility and stability. This study aimed to compare the safety and pharmacokinetic properties of the currently available and newly developed parenteral formulation of artesunate in healthy Thai volunteers.

**Methods:**

This was an open-label, randomized, 4 periods, 4-treatments, 24-sequence, single-dose, cross-over study in 72 male and female healthy Thai volunteers. Frequent pharmacokinetic samples were collected in all volunteers at each dose occasion. Observed concentration–time profiles were analysed with a non-compartmental approach followed by a bioequivalence evaluation.

**Results:**

Both intramuscular and intravenous administrations of the new parenteral formulation of artesunate were safe and well-tolerated, with no additional safety signals compared to the currently used formulation. The pharmacokinetic properties of artesunate and its active metabolite, dihydroartemisinin, were well-characterized, and showed rapid conversion of artesunate into dihydroartemisinin. Intramuscular administration of the newly formulated artesunate resulted in almost complete bioavailability of dihydroartemisinin. The pharmacokinetic properties were similar between the old and new formulation.

**Conclusions:**

The new and more easily prepared formulation of artesunate was safe and well-tolerated, with similar pharmacokinetic properties compared to the currently used formulation. Dihydroartemisinin, the active metabolite responsible for the majority of the anti-malarial effect, showed equivalent exposure after both intravenous and intramuscular administration of artesunate, suggesting that both routes of administration should generate comparable therapeutic effects.

*Trial registration*: The study was registered to clinicaltrials.gov (#TCTR20170907002).

**Supplementary Information:**

The online version contains supplementary material available at 10.1186/s12936-024-05085-9.

## Background

In 2022, there were an estimated 249 million cases of malaria worldwide, leading to 608,000 deaths. Children under the age of 5 accounted for 76% of the total malaria-related deaths [1]. Artesunate belongs to the artemisinin class of anti-malarial drugs which are the most potent and rapidly acting drugs currently available for the treatment of malaria. Artesunate kills all erythrocytic stages of the malaria parasite, including the young ring stage parasites, as well as late stage gametocytes responsible for malaria transmission [2, 3]. Artesunate is available in different formulations that allow for oral, rectal and parenteral administration. Oral formulations of artemisinin derivatives in combination with a longer acting partner drug (artemisinin-based combination therapy; ACT) are the first-line treatment of uncomplicated malaria recommended by the World Health Organization (WHO) [4]. Artesunate is the only artemisinin derivative that can be dissolved in water and, therefore, can be administered intravenously (IV) or intramuscularly (IM) to ensure rapid resolution of severe malaria and prevent death [4–6]. The WHO recommends dosing of 2.4 mg/kg (3.0 mg/kg in children < 20 kg) every 12 h for at least 24 h and until the patient is able to tolerate oral medication [4, 7, 8]. Once a patient has received at least 24 h of parenteral therapy and can tolerate oral therapy, the treatment is completed with a standard 3-day course of an ACT.

Parenteral administration of artesunate is associated with very high initial drug concentrations, which decline rapidly, resulting in a typical elimination half-life of less than 15 min [9]. After administration, artesunate is rapidly converted into its active metabolite, dihydroartemisinin, by esterase in the blood and by cytochrome P450 (CYP) 2A6 [10]. Dihydroartemisinin concentrations peak within 25 min post-dose, and dihydroartemisinin is eliminated with a terminal elimination half-life of 30–60 min [9]. Dihydroartemisinin is glucuronidated by UDP-glucuronosyltransferase (UGT) 1A9 and 2B7 in the gastrointestinal tract and liver into inactive glucuronide metabolites [11]. Both artesunate and dihydroartemisinin pharmacokinetics have shown considerable within- and between-patient variability [7, 12].

Artesunate and dihydroartemisinin have equivalent anti-malarial effects, but dihydroartemisinin accounts for most of the anti-malarial treatment effect because of its greater exposure compared to artesunate [13]. The efficacy of anti-malarial drugs depends on several factors, including parasite susceptibility, drug quality, drug adherence, dosing regimen, and the pharmacokinetic drug properties in the group of patients being treated. Severe malaria is a life-threatening disease and often accompanied by high parasite burden at presentation. Thus, it is critical to achieve maximum antiparasitic efficacy as soon as possible in the treatment of severe malaria.

Appropriate preparation of the parenteral artesunate formulation is crucial in order to ensure therapeutic effectiveness. Artesunate is unstable in neutral solution, so it has to be kept as anhydrous powder of artesunic acid, and the currently available injectable formulation of artesunate must be dissolved in 5% (w/v) sodium bicarbonate solution to form artesunate and then be diluted with physiological saline solution immediately before administration. However, it is difficult and time-consuming to prepare artesunate satisfactory in this two-step procedure prior to injection. If the solution is cloudy or a precipitate is present, the preparation must be discarded. This preparation process is not easily implemented in the field, and could lead to unsafe drug administration (e.g. clotting issues and inflammatory responses associated with injection of precipitated drug solutions). Injection of precipitated drug solutions can also lead to variations in dosing and potentially under-dosing of this life-saving treatment. A novel solvent for rapid and stable reconstitution of artesunate was developed recently by Guilin Pharmaceutical Co., Ltd, showing improved solubility and overall stability. This new injectable formulation received WHO pre-qualification in June 2023 [14]. The aim of this study was to compare the safety and pharmacokinetic properties of artesunate and dihydroartemisinin after IV and IM administration of the currently available and the new parenteral formulation of artesunate in healthy Thai volunteers.

## Methods

### Study participants and design

A total of 72 healthy male and female volunteers were enrolled in an open-label, randomized, 4 periods, 4-treatments, 24-sequence, single-dose, cross-over study in the Hospital of Tropical Diseases, Faculty of Tropical Medicine, Mahidol University, Bangkok, Thailand. Volunteers were screened according to the pre- defined inclusion/exclusion criteria.

Volunteers were enrolled only if their health was fully verified, including verification that serum biochemistry and haematology parameter values were within pre-defined normal ranges. The inclusion criteria included being healthy, male or female, aged between 18 and 55 years old with body mass index of 18–25 kg/m^2^, no evidence of underlying disease, normal electrocardiogram with Fridericia-corrected QT (QTcF) and Bazett-corrected QT (QTcB) intervals < 450 ms, not pregnant, agrees to using effective contraceptive methods during the study period, willingness to participate in the study, and provision of a signed written inform consent.

Volunteers were excluded if they had history of (or suspected of) hypersensitivity to artesunate or any ingredients in the study drug preparation, history of increased risk for bleeding (e.g. history of abnormal bleeding after minor injury or prolonged haematoma following intramuscular injection, thrombocytopenia (platelet < 150,000 per µl), abnormal coagulation (prothrombin time or partial thromboplastin time > upper normal limit or > 1.5 of the international normalized ratio), received any form of anti-coagulant within 14 days prior to the study drug administration or planned to receive any form of anti-coagulant while participating in the study, raised transaminase enzymes (AST or ALT 1.5 × upper normal limit), or were HIV, HBV or HCV positive. Additional exclusion criteria were a history of cardiac disease or arrhythmias, family history of sudden cardiac death, administration of a drug with known QT-prolongation properties (e.g. mefloquine, lumefantrine, chloroquine, quinine, and piperaquine) in the preceding 3 months prior to the starting of the study, participation in a clinical trial or receiving any drug or a new chemical entity within 30 days, 5 half-lives or twice the duration of the biological effect (whichever is longer) prior to the first dose of study medication, donated > 300 mL of blood within 30-day prior to the study, history or suspected substance abuse or dependence, and unwilling to abstain from alcohol consumption in the 48 h prior to drug administration.

Volunteers were screened within 14 days before the first drug administration. They were hospitalized a day before administration of study drug in each dosing period. There were four study drug regimens, including test formulation by IV injection (TIV), test formulation by IM injection (TIM), reference formulation by IV injection (RIV) and reference formulation by IM injection (RIM). All volunteers were randomly assigned to one of the 24 possible treatment sequences of the four study regimens in the four different study periods (supplement Table S1), with a maximum allocation of three volunteers to each specific treatment sequence. The randomized order of receiving each study drug (i.e. treatment sequence) was generated using Stata statistical software, version 14.0. There were 3 washout periods of at least 7 days between dosing events.

### Sample size calculation

This study was designed as a cross-over bioequivalence trial. Bioequivalence was declared if the exposure parameters were equivalent in the test and reference formulations. The US Food and Drug Administration (FDA) standard bioequivalence acceptance reference of 0.8–1.25 was used in the sample size calculation [15]. The expected mean ratio of test/reference formulation was assumed to be 1.00, and the study sample size calculation was based on 90% power (beta = 0.90) at 5% significance level (alpha = 0.05). From a previous population pharmacokinetic study, the within-patient variability was estimated to 42% (CV%; coefficient of variation (CV) × 100%), resulting in a within-patient variance of 0.16 (i.e. σ^2^ = *ln*(1 + CV^2^)). Based on these parameters, the sample size was calculated to be 72 individuals [16, 17]. Discontinued volunteers were replaced to maintain a total sample size of 72.

### Study drugs and administration

Both the new parenteral formulation of artesunate (test formulation) and the currently available parenteral formulation of artesunate (reference formulation) were provided by the Guilin Pharmaceutical Co., Ltd. (Guilin). Both preparations were stored below 30 °C and protected from light. The reconstitution of study drug and administration was performed according to a pre-defined approved work instruction. In brief, the reconstitution was done under aseptic conditions in a temperature-controlled room (< 30 °C) by trained and qualified personnel. The reconstituted artesunate solution was injected within 1 h of preparation.

A total dose of 2.4 mg/kg of study drug was administered as a single dose in each treatment arm (TIM, TIV, RIM, and RIV), and the total amount of artesunate administered (i.e. mg dose) was identical within a volunteer across all dosing occasions. The total injection volume was calculated for each dose administration based on the formula below, with a rounding precision of 0.2 ml.

$Injection \; volume \left(ml\right)=body weight (kg)\times \frac{2.4 (mg/kg)}{20 (mg/ml)}$ Test IV; Test IM; Reference IM.

$$Injection volume \left(ml\right)=body weight (kg)\times \frac{2.4 (mg/kg)}{10 (mg/ml)}$$ Reference IV.

Reconstituted artesunate solutions were injected slowly over 1–2 min. IM administration was performed in the anterior thigh, and if the total volume exceeded 10 ml, the volume was divided equally and injected in both thighs. The total fluid intake was restricted to a maximum of 3 L per day during the dosing period. In addition, volunteers refrained from grapefruit, illicit drugs, tea, coffee, caffeinated beverages, alcohol, sedatives, hypnotics, and stabilizers throughout the study period. Alcohol consumption was not allowed within 48 h prior to study drug administration and throughout the study.

### Pharmacokinetic sampling

Blood samples were obtained by IV cannula for the duration of sampling, and normal saline solution was used to flush the cannula after each sample. An initial volume of < 1.0 ml of blood was collected and discarded prior to collection of each whole blood sample to ensure that the saline solution did not dilute the samples. Pharmacokinetic samples (3 ml of blood) were collected before drug administration (0 h, pre-dose) and at 5 min, 15 min, 30 min, 45 min, 1 h, 1.5 h, 2 h, 3 h, 4 h, 6 h, 8 h, 10 h, 12 h and 24 h after drug administration, resulting in a total of 15 blood samples collected from each volunteer at each of the 4 treatment occasions. Blood samples were collected into pre-chilled fluoride-oxalate tubes, placed on ice and centrifuged within 15 min of collection to obtain plasma (i.e. centrifuged at 4 °C and 2000 × g for 7 min). Plasma was transferred into cryovial within 15 min of centrifugation. All plasma samples were stored temporarily at − 20 °C and transferred to − 80 °C within 48 h and stored until drug concentration analysis.

Drug measurements were performed at the Clinical Pharmacology Laboratory (ISO15189; ISO15190), Mahidol Oxford Tropical Medicine Research Unit (MORU), Faculty of Tropical Medicine, Bangkok, Thailand. A validated LC–MS/MS bioanalytical method was used to quantify the drug concentrations of artesunate and dihydroartemisinin in plasma [18]. All quality control samples were within regulatory acceptance limits (± 15%CV).

### Safety

History, physical examination, vital signs, 12-lead ECG measurements, and laboratory evaluations (biochemistry, haematology, electrolytes and urine examination) were conducted a day before drug administration and before discharge at each study occasion. Adverse events (AE) were monitored after drug administration and throughout the study period. AEs were assessed and graded following the Division of AIDS (DAIDS) Table for Grading the Severity of Adult and Paediatric Adverse Events, version 2.0, November, 2014 [19].

ECG measurements (Nihon Kohden ECG-1250 Cardiofax S, Tokyo, Japan) were recorded before the drug administration (pre-dose) and just before blood sampling at 10 min, 30 min, 1 h, 2 h, and 24 h. All ECGs were read manually and QT-intervals were corrected for heart rate using the Bazett correction (QTcB) and the Fridericia correction (QTcF). The most appropriate correction factor was determined by ordinary linear regression of corrected QT and ventricular rate. Derived QT-prolongation (ΔQT), based on pre-dose QT reading vs post-dose reading, was evaluated by ordinary linear regression of ΔQT and drug concentrations. All volunteers who received at least one dose of study drug were included in the safety analysis. Count data were summarized using frequency counts and percentages. The incidence of AEs was tabulated and reviewed for potential clinical importance. The frequency of AEs was compared between treatment arms using the Fisher’s exact test at a significance level of 5%.

### Pharmacokinetic analysis

Volunteers who received the study drug as per protocol (i.e. those who completed all drug administrations) were included in the pharmacokinetic analysis. Individual pharmacokinetic parameters of artesunate and dihydroartemisinin were calculated after each dose administration using a non-compartmental approach in Phoenix 64 v.8.1 (Certara, USA). Drug concentration measurements below the lower limit of quantification were ignored in the analysis. Total exposure up to the last measured concentration (AUC_LAST_) was calculated using the linear trapezoidal method for ascending concentrations and the logarithmic trapezoidal method for descending concentrations. The terminal elimination half-life (t_½_) was estimated by the slope (_λZ_) of the best-fit log-linear regression of the observed concentrations in the terminal elimination phase. Drug exposure was extrapolated from the last observed concentration to time infinity by C_LAST/λZ_ for each individual volunteer to compute total drug exposure (AUC_∞_). The peak concentration (C_MAX_) and time to peak concentration (T_MAX_) were taken directly from the observed data. Elimination clearance (CL) and volume of distribution (V) were computed individually. Pharmacokinetic parameters were summarized and stratified by group.

An analysis of variance (ANOVA) was carried out on the log-transformed pharmacokinetic exposure parameters (C_MAX_, AUC_LAST_, and AUC_∞_) to assess the bioequivalence of the drug formulations. If the administered doses of the test and reference formulation deviated with more than 5% within a volunteer, the above analysis was carried out with dose-normalized parameter values. Bioequivalence was assumed if the 90% CIs of the log-transformed ratio (Test/Reference) of exposure parameters fell within 80–125% [15, 20]. As a secondary analysis the IM bioequivalence of artesunate and dihydroartemisinin exposures were evaluated, comparing the IM test formulation and the IV test formulation. Artesunate and dihydroartemisinin were evaluated separately, but also by adding the molar concentrations of artesunate and dihydroartemisinin at each time-point for each individual before the pharmacokinetic analysis. The combined exposure analysis was conducted as both compounds have equipotent anti-malarial efficacy, but somewhat different pharmacokinetic profiles, in order to evaluate the relevant pharmacokinetic profile linked to treatment efficacy.

## Results

**Fig. 1:**
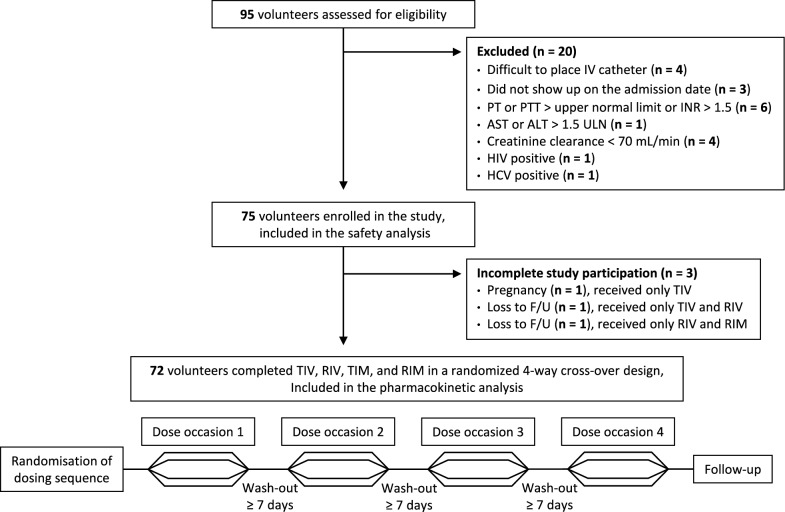
Study design and enrolment. ALT, alanine transaminase; AST, aspartate aminotransferase; F/U, follow up; HCV hepatitis C virus; HIV, human immunodeficiency virus; INR, international normalized ratio; IV, intravenous; n, number of volunteers; PT, prothrombin time; PTT, partial prothrombin time; RIM, reference formulation administered intramuscularly; RIV, reference formulation administered intravenously; TIM, test formulation administered intramuscularly; TIV, test formulation administered intravenously; and ULN, upper limit of normal

A total of 95 volunteers were screened, and 20 failed the screen, resulting in 40 male and 35 female healthy volunteers enrolled into the study (Table 1 and Fig. 1). All volunteers were Thai. The 3 volunteers who did not complete all dose occasions were included in the safety analysis but not in the PK analysis. In conclusion, 74 volunteers received TIV, 72 volunteers received TIM, 74 volunteers received RIV, and 73 volunteers received RIM.

**Table 1:** Summary of volunteer demographic variables

Demographics	Baseline enrolment (safety analysis)	Complete drug administration (pharmacokinetic analysis)
Total volunteers	75	72
Male, n (%)	40 (53%)	39 (54%)
Female, n (%)	35 (47%)	33 (46%)
Age (years)	34 (21–54)	35 (21–54)
Body weight (kg)	59 (44–82)	59 (44–82)
Height (cm)	164 (147–183)	164 (147–183)
BMI (kg/m^2^)	22.2 (18.1–24.9)	22.3 (18.5–24.9)

All variables are presented as median (min–max range) if not otherwise stated

### Safety analysis

Both formulations and routes of administration were well tolerated, and no severe adverse events were reported. There were a total of 98 AEs in 46 volunteers, out of 75 volunteers receiving a total of 293 treatment doses (33.4%) across the 4 treatment arms (Table 2). Almost all AEs were considered mild, except for 1 volunteer with moderate pain at the injection site after receiving the reference formulation as an IM injection. Among these 98 AEs, 48 AEs in 31 volunteers were considered related to the study drug, and all of them were reported in all treatment arms. The other, 50 AEs were not considered related to the study drug, and they occurred across all 4 treatment arms. 97 out of the 98 AEs were of mild severity and only one AE was of moderate severity (pain at the injection site).

**Table 2:** Summary of adverse events

Adverse event	Treatment arm				
	Test IV (n = 74)	Reference IV (n = 74)	Test IM (n = 72)	Reference IM (n = 73)	Total treatments (n = 293)
Total adverse events	27	29	15	27	98
Bitter taste	14 (18.9%)	13 (17.6%)	0	0	27 (9.2%)
Pain at injection site	0	0	5 (6.9%)	13 (17.8%)	18 (6.1%)
Common cold	1 (1.4%)	3 (4.1%)	5 (6.9%)	1 (1.4%)	10 (3.4%)
Headache	1 (1.4%)	2 (2.7%)	1 (1.4%)	1 (1.4%)	5 (1.7%)
Rhinorrhoea	3 (4.1%)	1 (1.4%)	0	1 (1.4%)	5 (1.7%)
Diarrhoea	0	1 (1.4%)	0	2 (2.7%)	3 (1.0%)
Nausea	2 (2.7%)	1 (1.4%)	0	0	3 (1.0%)
Back pain	0	1 (1.4%)	0	1 (1.4%)	2 (0.7%)
Dizziness	1 (1.4%)	0	1 (1.4%)	0	2 (0.7%)
Metallic taste	1 (1.4%)	1 (1.4%)	0	0	2 (0.7%)
Sore throat	0	0	1 (1.4%)	1 (1.4%)	2 (0.7%)
Other	4 (5.4%)	6 (8.1%)	2 (2.8%)	7 (9.6%)	19 (6.5%)

All values are given as number of adverse events (%)

The most common AE was bitter taste (27 out of 98) that occurred only in IV arms (RIV and TIV) and pain at injection site (18 out of 98) occurring only in IM arms (RIM and TIM). All of these AEs were transient and returned to normal within 24 h of drug administration. There were no clinically relevant findings in laboratory chemistry and liver/kidney functions. Two volunteers had mild (Grade 1) haematological AEs of low haemoglobin (1 volunteer in RIV, 1 volunteer in TIV), considered not related to study treatment. Seven volunteers reported high systolic blood pressures and 3 reported high diastolic blood pressures after drug administration, but all of these AEs were transient, not clinically significant, and returned to baseline shortly after study drug administration. There were no uncorrected or corrected QT intervals measured above 500 ms in any volunteers during the study (Fig. S1). Fridericia correction provided the best correction for heart rate with no substantial residual trend in QTcF vs heart rate, as compared to Bazett showing a clear tendency of over-correction (Fig. S2). No ΔQTcF above 60 ms was reported during any of the 293 separate dosing events, and there was no correlation between ΔQTcF and drug concentration (Fig. S2).

### Pharmacokinetic analysis

**Fig. 2:**
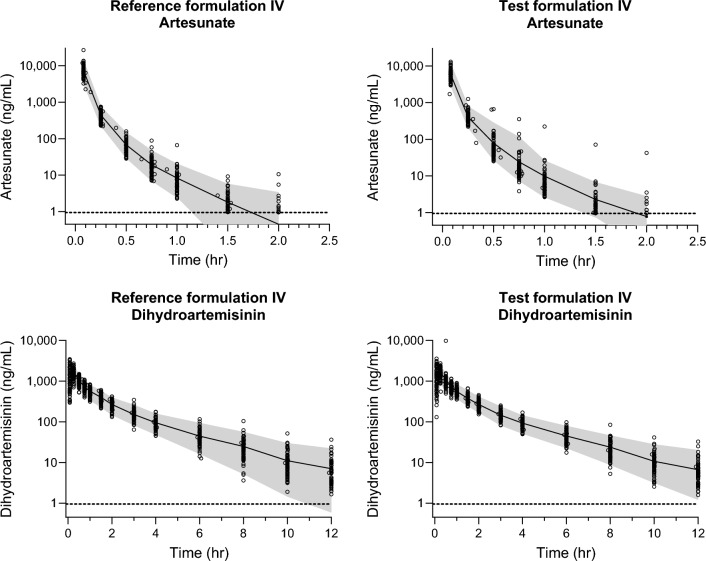
Pharmacokinetic concentration–time profiles of artesunate (left panel) and dihydroartemisinin (right panel), after IV administration of reference (upper panel) and test (lower panel) formulations. The open circles show individual drug measurements, and the solid lines and shaded areas show the average and 95% prediction interval of measured drug concentrations at each sampling time

**Fig. 3:**
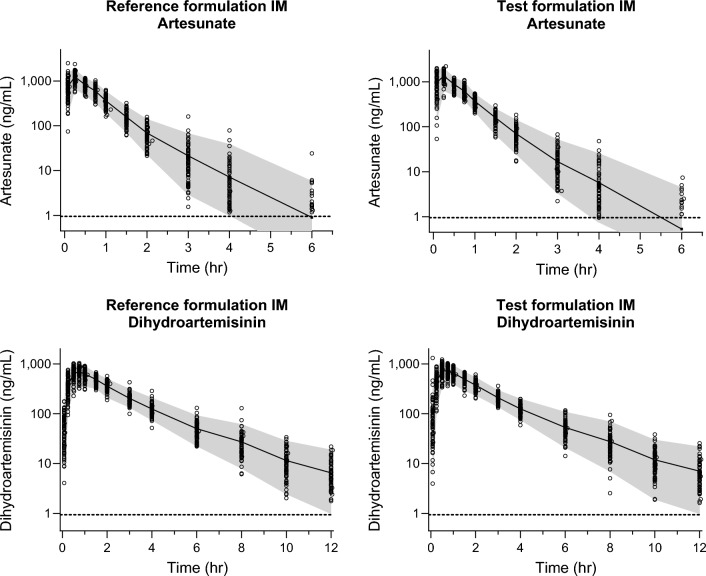
Pharmacokinetic concentration–time profiles of artesunate (left panel) and dihydroartemisinin (right panel), after IM administration of reference (upper panel) and test (lower panel) formulations. The open circles show individual drug measurements, and the solid lines and shaded areas show the average and 95% prediction interval of measured drug concentrations at each sampling time

All volunteers who received all drug administrations according to the protocol (2.4 mg/kg of TIV, RIV, TIM, and RIM) were included in the pharmacokinetic analysis. The pharmacokinetic properties of artesunate and dihydroartemisinin were evaluated separately, and also combined by adding the molar concentrations of artesunate and dihydroartemisinin at each time point for each volunteer before analysis (ART-DHA). The designed sampling schedule provided ideal data for a model-free analysis, resulting in complete pharmacokinetic profiles for all volunteers for both artesunate and dihydroartemisinin after IV and IM administration of the two formulations (Figs. 2 and 3). The pharmacokinetic parameters of artesunate and dihydroartemisinin are summarized and stratified by treatment arm in Tables 3 and 4. There were no apparent trends showing any substantial differences in the pharmacokinetic parameters for artesunate and dihydroartemisinin when comparing the test and reference formulation. As expected, there were substantial differences in the pharmacokinetic parameters of both artesunate and dihydroartemisinin when given IM compared to IV. Dose-normalization was not performed because all administered doses of artesunate (test and reference formulations) were within ± 5% for each patient.

**Table 3:** Pharmacokinetic parameters of artesunate, stratified by treatment arm

	Treatment arm			
Parameter	Reference IV (n = 72)	Test IV (n = 72)	Reference IM (n = 72)	Test IM (n = 72)
T_MAX_ (h)	–	–	0.25 (0.25–0.25)	0.25 (0.25–0.25)
C_MAX_ (ng/mL)	6705 (5365–8468)	5755 (4405–6773)	1230 (1020–1473)	1400 (1130–1665)
AUC_∞_ (h × ng/mL)	689 (590–886)	610 (505–757)	998 (847–1122)	1073 (908–1178)
AUC_LAST_ (h × ng/mL)	688 (589–886)	610 (504–756)	993 (844–1120)	1071 (907–1175)
t_1/2_ (h)	0.21 (0.18–0.26)	0.20 (0.15–0.22)	0.46 (0.37–0.62)	0.43 (0.37–0.53)
CL (L/h)	198 (162–252)	240 (185–284)	148 (130–161)	140 (123–159)
V (L)	60.7 (46.8–75.6)	64.0 (54.6–77.4)	102 (79.5–132)	86.9 (69.8–115)
F (%)	–	–	135 (117–171)	168 (132–206)

AUC_LAST_ is the area under the drug concentration–time curve from time zero to the last measurable drug concentration; AUC_∞_ is the area under the drug concentration–time curve from time zero to infinity; CL is the drug elimination clearance; C_MAX_ is the maximum observed plasma concentration; F is the absolute bioavailability; T_MAX_ is the time to maximum plasma concentration; t_1/2_ is the terminal elimination half-life; V is the apparent volume of distribution. Absolute IM bioavailability was calculated by dividing the individual total exposure (AUC_∞_) to that after IV administration for each volunteer. All values are presented as median (inter-quartile range)

**Table 4:** Pharmacokinetic parameters of dihydroartemisinin, stratified by treatment arm

	Treatment arm			
Parameter	Reference IV (n = 72)	Test IV (n = 72)	Reference IM (n = 72)	Test IM (n = 72)
T_MAX_ (h)	0.25 (0.08–0.25)	0.25 (0.08–0.25)	0.75 (0.50–0.75)	0.75 (0.50–0.75)
C_MAX_ (ng/mL)	1735 (1388–2140)	1705 (1380–2245)	749 (641–832)	760 (643–901)
AUC_∞_ (h × ng/mL)	1983 (1712–2248)	1929 (1682–2188)	1715 (1483–1924)	1846 (1540–2048)
AUC_LAST_ (h × ng/mL)	1968 (1704–2238)	1904 (1667–2170)	1702 (1466–1891)	1833 (1525–2042)
t_1/2_ (h)	1.88 (1.65–2.17)	1.83 (1.61–2.13)	1.65 (1.47–1.89)	1.72 (1.53–1.92)
CL (L/h)	52.5 (46.8–62.8)	54.7 (45.4–64.4)	62.4 (54.6–73.3)	59.2 (50.5–72.4)
V (L)	143 (126–174)	145 (118–174)	152 (128–183)	147 (122–168)
F (%)	–	–	87.0 (80.2–93.2)	92.6 (85.4–101)

AUC_LAST_ is the area under the drug concentration–time curve from time zero to the last measurable drug concentration; AUC_∞_ is the area under the drug concentration–time curve from time zero to infinity; CL is the drug elimination clearance; C_MAX_ is the maximum observed plasma concentration; F is the absolute bioavailability; T_MAX_ is the time to maximum plasma concentration; t_1/2_ is the terminal elimination half-life; V is the apparent volume of distribution. Absolute IM bioavailability was calculated by dividing the individual total exposure (AUC_∞_) to that after IV administration for each volunteer. All values are presented as median (inter-quartile range)

**Fig. 4:**
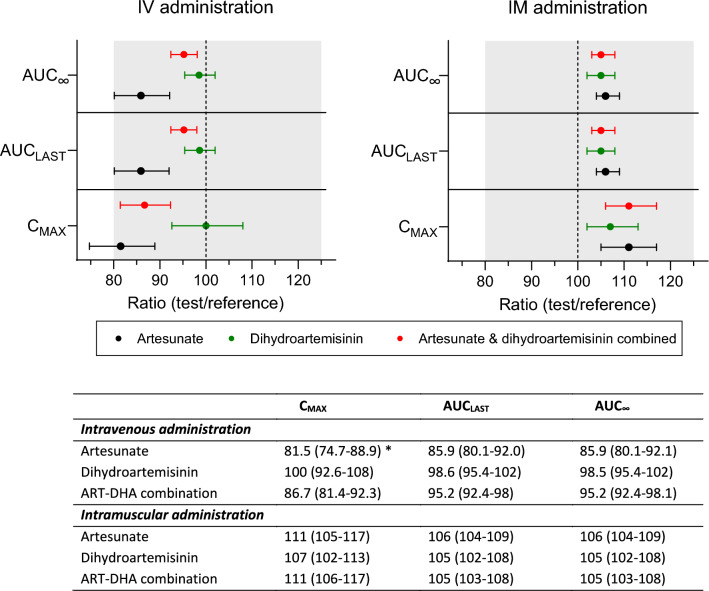
Bioequivalence of test formulation, compared to reference formulation, after IV and IM administration. Bioequivalence parameters are presented as the geometric mean ratio between the test and reference formulation (90% confidence interval). C_MAX_ is the maximum concentration; AUC_LAST_ is the area under the concentration–time curve from time zero to the last measurable observation; AUC_∞_ is the area under the concentration–time curve from time zero to infinity; ART-DHA is the combined molar drug measurement of artesunate and dihydroartemisinin. The shaded area in the plot shows the 80–125% criterion for bioequivalence. * Indicates that the criterion for bioequivalence was not fulfilled, i.e. the CI 90% of the ratio (test/reference) was not contained within 80–125%

Overall, the two drug formulations exhibited similar exposure and demonstrated bioequivalence, except for the maximum concentration of artesunate that was somewhat lower for the test formulation compared to the reference formulation (Fig. 4). All parameters for the main metabolite, dihydroartemisinin and for the evaluation of the combined exposure to parent and metabolite demonstrated bioequivalence. There was no significant (p > 0.05) sequence effect identified in the bioequivalence analysis.

## Discussion

### Safety

The IM or IV administration of the new parenteral formulation or the currently used parenteral formulation of AS was generally safe and well tolerated in this four-sequence crossover treatment in healthy volunteers. All AEs were mild and transient, except for 1 volunteer with moderately severe pain at the injection site after receiving IM reference formulation, and none of the reported AEs resulted in discontinuation from the study. The most common AE was bitter taste, reported in both IV arms at an equal frequency (18.9% vs 17.6%). Artesunate has been shown to attenuate airway resistance in animal models via bitter taste receptor-dependent calcium signalling [21] and artesunate and dihydroartemisinin concentrations in saliva have been reported to be directly proportional to that in plasma in patients with metastatic breast cancer [22]. This dose-dependent interaction might suggest that the very high drug concentrations, associated with IV administration, are needed to generate the bitter/metallic taste reported here. Pain at the injection site was the second most common AE, reported only after receiving IM administration, and was more commonly reported with the reference formulation (17.8%) compared to the test formulation (6.9%). This could be due to the larger injection volume associated with the reference formulation or the formulation itself. No QT-prolongation (ΔQTcF > 60 ms) were reported in any of the 293 dose occasions and there was no association between drug concentration and QT-prolongation. There were no deaths or other SAEs reported with either formulation of AS. All AEs reported in laboratory test and vital signs were transient, and their values returned to baseline shortly after completion of study drug administration. Thus, the new parenteral formulation of AS showed no additional safety signals compared with the currently used parenteral formulation.

### Pharmacokinetic analysis

Complete concentration–time profiles of both artesunate and dihydroartemisinin were achieved by the frequent blood sampling conducted here, enabling an unbiased model-independent analysis for each treatment occasion in all volunteers completing the four treatment arms. To the best of our knowledge, this is the most detailed pharmacokinetic evaluation of parenteral artesunate in the literature. The pharmacokinetic parameters presented here for artesunate and dihydroartemisinin were similar to those reported in previously published studies [12, 23–25]. Since both test and reference formulations were aqueous-based, their bioavailability was assumed to be 100% after IV administration, with an immediate presentation of artesunate in the systemic circulation, resulting in very high peak concentrations of artesunate. Artesunate was rapidly metabolized to dihydroartemisinin, peaking at approximately 15 min after IV administration and 45 min after IM administration. Thus, the absorption rate of artesunate from the injection site at the anterior thigh, was slower than the true elimination rate of artesunate seen with IV administration, resulting in absorption-limited kinetics (i.e., flip-flop) after IM administration. This can be seen clearly by a substantially shorter terminal elimination half-life of artesunate after IV compared to IM administration (0.20 *vs*. 0.44 h). The practical implications are an almost fivefold lower peak concentration, but a 61% larger total drug exposure to artesunate when administering an identical dose IM compared to IV. The larger exposure seen after IM administration also resulted in an absolute bioavailability of > 100% compared to IV administration. However, this has little clinical significance as dihydroartemisinin, which is the main driver associated with anti-malarial efficacy, does not show formation-rate limited kinetics after IM and IV dosing. Thus, the metabolism of artesunate into dihydroartemisinin is faster than the elimination of dihydroartemisinin, irrespectively of route of administration. The overall absolute bioavailability of dihydroartemisinin was high after intramuscular administration (average bioavailability of 90.9%), suggesting that intramuscular administration of artesunate should not compromise treatment efficacy compared to intravenous administration.

Observed peak concentrations of artesunate were lower after IV administration of the test formulation, compared to the reference formulation, resulting in the lower bound of the 90% confidence interval to drop below 80%. However, all other exposure parameters for artesunate, dihydroartemisinin and the combined drug measurements of artesunate and dihydroartemisinin demonstrated bioequivalence after both IV and IM administration. The very rapid biotransformation of artesunate to dihydroartemisinin results in a typical terminal elimination half-life of less than 15 min. This rapid conversion combined with an IV injection of test and reference formulation directly into the systemic circulation, might have resulted in early sampling time points (i.e., 5-min and 15-min post-dose) failing to describe completely the initial concentration–time profile of artesunate. This might explain why bioequivalence could not be concluded for artesunate peak concentration when administered IV. However, these slightly lower observed peak concentrations of artesunate have no clinical significance, either in terms of safety or therapeutic effectiveness. The minimal inhibitory concentration (MIC) of artesunate is more than 1000-fold lower than these observed concentrations [26] and the therapeutic efficacy is more likely related to time-above-MIC or total drug exposure, rather than peak concentrations. Furthermore, these two pharmacodynamic drivers are highly dependent on dihydroartemisinin due to the considerably longer terminal elimination half-life (0.20 *vs*. 1.86 h after IV administration) and the approximately threefold higher total exposure to dihydroartemisinin compared to artesunate (646 *vs*. 1970 h × ng/mL after IV administration).

The main limitation of this study is that artesunate and dihydroartemisinin were evaluated in healthy volunteers, whom might have different pharmacokinetic properties compared to patients with severe malaria. However, it is unlikely that patients would show a substantial difference in pharmacokinetic properties between the new, more easily prepared formulation and the reference formulation when this was not seen in a detailed healthy volunteer study.

## Conclusions

In conclusion, this healthy volunteer trial demonstrated bioequivalence of a newly developed more easily prepared, and pre-qualified parenteral formulation of artesunate when compared to the currently used parenteral formulation. Both the active metabolite, dihydroartemisinin, responsible for most of the therapeutic efficacy and an evaluation of combined parent and metabolite exposure showed bioequivalence. Thus, it is assumed that this novel simplified and more stable parenteral formulation of artesunate would result in equivalent therapeutic efficacy in patients, compared to currently available formulations. Both IM or IV administration of this new formulation was safe and well-tolerated, and showed no additional safety signals compared to the reference formulation.

## Supplementary Information

Additional file 1.

## Data Availability

Deidentified individual participant data will be available after publication to applicants who provide a sound proposal to the Mahidol Oxford Tropical Medicine Research Unit Data Access Committee. They can contact the first author in the first instance.

## Abbreviations

ACT: Artemisinin-based combination therapy
WHO: World Health Organization
IV: Intravenous
IM: Intramuscular
CYP: Cytochrome P450
UGT: UDP-glucuronosyltransferase
TIV: Test formulation by IV injection
TIM: Test formulation by IM injection
RIV: Reference formulation by IV injection
RIM: Reference formulation by IM injection
FDA: US Food and Drug Administration
CV: Coefficient of variation
MORU: Mahidol Oxford Tropical Medicine Research Unit
AE: Adverse events
AUC: Area under the concentration–time curve
t_½_: Terminal elimination half-life
C_MAX_: Maximum concentration
T_MAX_: Time to maximum concentration
CL: Elimination clearance
V: Volume of distribution
ANOVA: Analysis of variance
ART: Artesunate
DHA: Dihydroartemisinin
MIC: Minimal inhibitory concentration

## References

[1] WHO. World malaria report 2023: tracking progress and gaps in the global response to malaria. Geneva: World Health Organization; 2023. p. 2023.

[2] Piyaphanee W, Krudsood S, Tangpukdee N, Thanachartwet W, Silachamroon U, Phophak N, et al. Emergence and clearance of gametocytes in uncomplicated *Plasmodium falciparum* malaria. Am J Trop Med Hyg. 2006;74:432–5. 16525102

[3] White NJ. Malaria parasite clearance. Malar J. 2017;16:88. 28231817 10.1186/s12936-017-1731-1 PMC5324257

[4] WHO. Guidelines for malaria. Geneva: World Health Organization; 2023.

[5] Dondorp A, Nosten F, Stepniewska K, Day N, White N. South East Asian Quinine Artesunate Malaria Trial Group. Artesunate versus quinine for treatment of severe falciparum malaria: a randomised trial. Lancet. 2005;366:717–25. 16125588 10.1016/S0140-6736(05)67176-0

[6] Dondorp A, Fanello C, Hendriksen IC. Artesunate versus quinine in the treatment of severe falciparum malaria in African children (AQUAMAT): an open-label, randomised trial. Lancet. 2010;376:1647–57. 21062666 10.1016/S0140-6736(10)61924-1 PMC3033534

[7] Zaloumis SG, Tarning J, Krishna S, Price RN, White NJ, Davis TM, et al. Population pharmacokinetics of intravenous artesunate: a pooled analysis of individual data from patients with severe malaria. CPT Pharmacometrics Syst Pharmacol. 2014;3:e145. 25372510 10.1038/psp.2014.43 PMC4259998

[8] Hendriksen IC, Mtove G, Kent A, Gesase S, Reyburn H, Lemnge MM, et al. Population pharmacokinetics of intramuscular artesunate in African children with severe malaria: implications for a practical dosing regimen. Clin Pharmacol Ther. 2013;93:443–50. 23511715 10.1038/clpt.2013.26 PMC3630454

[9] Morris CA, Duparc S, Borghini-Fuhrer I, Jung D, Shin C-S, Fleckenstein L. Review of the clinical pharmacokinetics of artesunate and its active metabolite dihydroartemisinin following intravenous, intramuscular, oral or rectal administration. Malar J. 2011;10:263. 21914160 10.1186/1475-2875-10-263 PMC3180444

[10] Li XQ, Björkman A, Andersson TB, Gustafsson LL, Masimirembwa CM. Identification of human cytochrome P(450)s that metabolise anti-parasitic drugs and predictions of in vivo drug hepatic clearance from in vitro data. Eur J Clin Pharmacol. 2003;59:429–42. 12920490 10.1007/s00228-003-0636-9

[11] Ilett KF, Ethell BT, Maggs JL, Davis TM, Batty KT, Burchell B, et al. Glucuronidation of dihydroartemisinin in vivo and by human liver microsomes and expressed UDP-glucuronosyltransferases. Drug Metab Dispos. 2002;30:1005–12. 12167566 10.1124/dmd.30.9.1005

[12] Kloprogge F, McGready R, Phyo AP, Rijken MJ, Hanpithakpon W, Than HH, et al. Opposite malaria and pregnancy effect on oral bioavailability of artesunate - a population pharmacokinetic evaluation. Br J Clin Pharmacol. 2015;80:642–53. 25877779 10.1111/bcp.12660 PMC4594700

[13] Newton PN, van Vugt M, Teja-Isavadharm P, Siriyanonda D, Rasameesoroj M, Teerapong P, et al. Comparison of oral artesunate and dihydroartemisinin antimalarial bioavailabilities in acute falciparum malaria. Antimicrob Agents Chemother. 2002;46:1125–7. 11897605 10.1128/AAC.46.4.1125-1127.2002 PMC127101

[14] WHO. Prequalification of medical products: M168. Geneva: World Health Organization; 2023.

[15] FDA. Statistical approaches to establishing bioequivalence guidance for industry. 2022. https://www.fda.gov/media/70958/download.

[16] Chow SC. Design and analysis of clinical trials: concepts and methodologies. 3rd ed. New York: John Wiley & Sons; 2013.

[17] Steven A. Sample size for clinical trials. 1st ed. New York: Chapman & Hall/CRC Taylor & Francis Group; 2009.

[18] Hanpithakpong W, Kamanikom B, Dondorp AM, Singhasivanon P, White NJ, Day NP, et al. A liquid chromatographic-tandem mass spectrometric method for determination of artesunate and its metabolite dihydroartemisinin in human plasma. J Chromatogr B Analyt Technol Biomed Life Sci. 2008;876:61–8. 18990614 10.1016/j.jchromb.2008.10.018

[19] DAIDS. Division of AIDS table for grading the severity of adult and pediatric adverse events. Clarification august 2009 (Version 10). 2004.

[20] FDA. Guidance for Industry: bioavailability and Bioequivalence Studies for Orally Administered Drug Products—General Considerations. 2003. https://www.fda.gov/media/88254/download.

[21] Wang Y, Wang A, Zhang M, Zeng H, Lu Y, Liu L, et al. Artesunate attenuates airway resistance in vivo and relaxes airway smooth muscle cells in vitro via bitter taste receptor-dependent calcium signalling. Exp Physiol. 2019;104:231–43. 30379382 10.1113/EP086824

[22] Ericsson T, Blank A, von Hagens C, Ashton M, Äbelö A. Population pharmacokinetics of artesunate and dihydroartemisinin during long-term oral administration of artesunate to patients with metastatic breast cancer. Eur J Clin Pharmacol. 2014;70:1453–63. 25248945 10.1007/s00228-014-1754-2

[23] Miller RS, Li Q, Cantilena LR, Leary KJ, Saviolakis GA, Melendez V, et al. Pharmacokinetic profiles of artesunate following multiple intravenous doses of 2, 4, and 8 mg/kg in healthy volunteers: phase 1b study. Malar J. 2012;11:255. 22853818 10.1186/1475-2875-11-255 PMC3468400

[24] Li Q, Cantilena LR, Leary KJ, Saviolakis GA, Miller RS, Melendez V, Weina PJ. Pharmacokinetic profiles of artesunate after single intravenous doses at 05, 1, 2, 4, and 8 mg/kg in healthy volunteers: a phase I study. Am J Trop Med Hyg. 2009;81:615–21. 19815876 10.4269/ajtmh.2009.09-0150

[25] Ward KW, Hardy LB, Kehler JR, Azzarano LM, Smith BR. Apparent absolute oral bioavailability in excess of 100% for a vitronectin receptor antagonist (SB-265123) in rat. II. Studies implicating transporter-mediated intestinal secretion. Xenobiotica. 2004;34:367–77. 15268981 10.1080/0049825042000205540a

[26] Brockman A, Price RN, van Vugt M, Heppner DG, Walsh D, Sookto P, et al. *Plasmodium falciparum* antimalarial drug susceptibility on the north-western border of Thailand during five years of extensive use of artesunate-mefloquine. Trans R Soc Trop Med Hyg. 2000;94:537–44. 11132385 10.1016/s0035-9203(00)90080-4 PMC4340572

