# Supplementary material for: Safety and pharmacokinetic properties of a new formulation of parenteral artesunate in healthy Thai volunteers
Source: Malar J. 2024 Oct 3;23:296. doi: 10.1186/s12936-024-05085-9 (PMC11450984; doi:10.1186/s12936-024-05085-9)
Supplement: Supplementary file 1 — Additional file 1. [file 12936_2024_5085_MOESM1_ESM.pdf]

Supplement Material

Safety and pharmacokinetic properties of a new formulation of parenteral artesunate in healthy Thai volunteers

Joel Tarning, Borimas Hanboonkunupakarn, Richard M. Hoglund, Kesinee Chotivanich, Mavuto Mukaka, Sasithon Pukrittayakamee, Nicholas P.J. Day, Nicholas J. White, Arjen M. Dondorp, Podjanee Jittamal

---

Table S1. All possible treatment sequences.

Sequence Number	Regimen 1	Regimen 2	Regimen 3	Regimen 4	Dosing Sequence
1	RIM	RIV	TIM	TIV	RIM-RIV-TIM-TIV
2	RIM	RIV	TIV	TIM	RIM-RIV-TIV-TIM
3	RIM	TIM	RIV	TIV	RIM-TIM-RIV-TIV
4	RIM	TIM	TIV	RIV	RIM-TIM-TIV-RIV
5	RIM	TIV	RIV	TIM	RIM-TIV-RIV-TIM
6	RIM	TIV	TIM	RIV	RIM-TIV-TIM-RIV
7	RIV	RIM	TIM	TIV	RIV-RIM-TIM-TIV
8	RIV	RIM	TIV	TIM	RIV-RIM-TIV-TIM
9	RIV	TIM	RIM	TIV	RIV-TIM-RIM-TIV
10	RIV	TIM	TIV	RIM	RIV-TIM-TIV-RIM
11	RIV	TIV	RIM	TIM	RIV-TIV-RIM-TIM
12	RIV	TIV	TIM	RIM	RIV-TIV-TIM-RIM
13	TIM	RIM	RIV	TIV	TIM-RIM-RIV-TIV
14	TIM	RIM	TIV	RIV	TIM-RIM-TIV-RIV
15	TIM	RIV	RIM	TIV	TIM-RIV-RIM-TIV
16	TIM	RIV	TIV	RIM	TIM-RIV-TIV-RIM
17	TIM	TIV	RIM	RIV	TIM-TIV-RIM-RIV
18	TIM	TIV	RIV	RIM	TIM-TIV-RIV-RIM
19	TIV	RIM	RIV	TIM	TIV-RIM-RIV-TIM
20	TIV	RIM	TIM	RIV	TIV-RIM-TIM-RIV
21	TIV	RIV	RIM	TIM	TIV-RIV-RIM-TIM
22	TIV	RIV	TIM	RIM	TIV-RIV-TIM-RIM
23	TIV	TIM	RIM	RIV	TIV-TIM-RIM-RIV
24	TIV	TIM	RIV	RIM	TIV-TIM-RIV-RIM

RIM = reference formulation administered intramuscularly; RIV = reference formulation administered intravenously; TIM = test formulation administered intramuscularly; TIV = test formulation administered intravenously. Volunteers were randomised to treatment sequence, with an equal distribution of all 24 individual sequences in 72 volunteers that completed all treatment periods (i.e. 3 volunteers per treatment sequence).

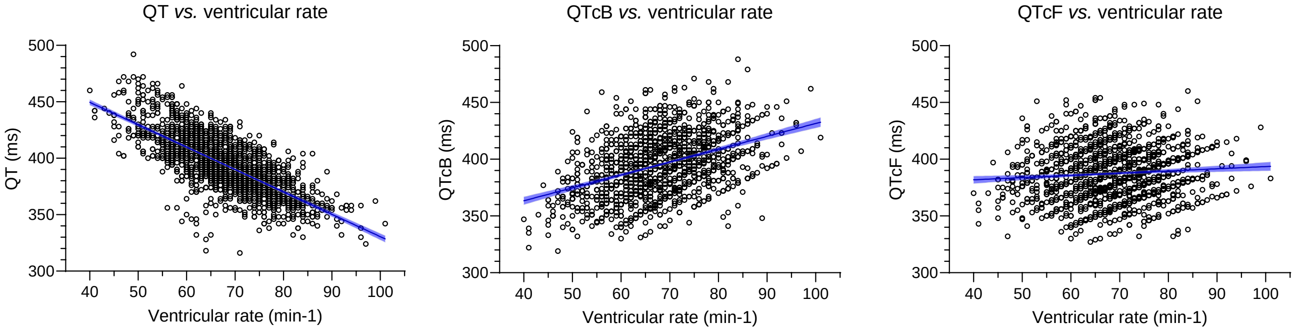

Figure S1. Uncorrected QT, Bazett-corrected QT (QTcB) and Fridericia-corrected QT (QTcF) plotted against ventricular rate. Open circles are observed data, solid blue lines are linear regressions of observed data, and the shaded blue area is the 95% confidence bands of the best-fit regression line.

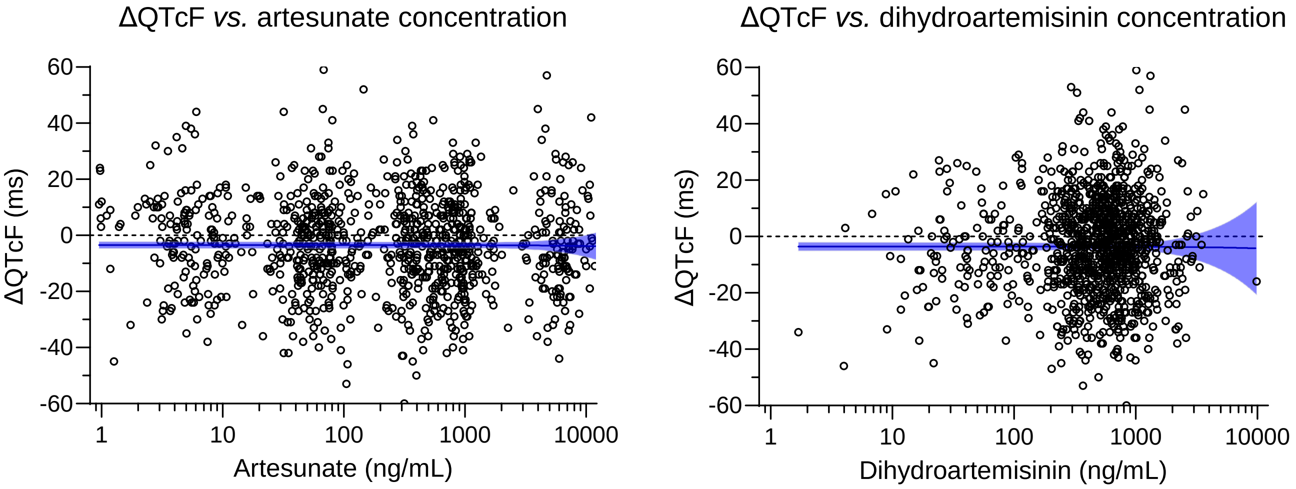

Figure S2. Fridericia-corrected QT (QTcF) plotted against drug concentrations. Open circles are observed data, solid blue lines are linear regressions of observed data, and the shaded blue area is the 95% confidence bands of the best-fit regression line.
